# KSR2-14–3-3ζ complex serves as a biomarker and potential therapeutic target in sorafenib-resistant hepatocellular carcinoma

**DOI:** 10.1186/s40364-022-00361-9

**Published:** 2022-04-25

**Authors:** Chao Gao, Si-wei Wang, Jia-cheng Lu, Xiao-qiang Chai, Yuan-cheng Li, Peng-fei Zhang, Xiao-yong Huang, Jia-bin Cai, Yi-min Zheng, Xiao-jun Guo, Guo-ming Shi, Ai-wu Ke, Jia Fan

**Affiliations:** 1 Institutes of Biomedical Sciences, Fudan University, Shanghai, China; 2 Key Laboratory of Carcinogenesis and Cancer Invasion, Department of Liver Surgery, Ministry of Education, Zhongshan Hospital, Liver Cancer Institute, Fudan University, Shanghai, China

**Keywords:** Hepatocellular carcinoma, KSR2, 14–3-3ζ, MAPK pathway, Sorafenib

## Abstract

**Background:**

Kinase suppressor of Ras 2 (KSR2) is a regulator of MAPK signaling that is overactivated in most hepatocellular carcinoma (HCC). We sought to determine the role of KSR2 in HCC pathogenesis.

**Methods:**

We tested the level of KSR2 in HCC tissues and cell lines by tissue microarray, qPCR, and western blotting. Functionally, we determined the effects of KSR2 on the proliferation, migration, and invasion of HCC cells through colony formation assays, scratch assays, transwell migration assays, and xenograft tumor models. Co-immunoprecipitation (co-IP) experiments were used to assess the interaction of phospho-serine binding protein 14–3-3ζ and KSR2, and the effects of this interaction on growth and proliferation of human HCC cells were tested by co-overexpression and knockdown experiments. Additionally, we used flow cytometry to examine whether the KSR2 and 14–3-3ζ interaction conveys HCC resistance to sorafenib.

**Results:**

KSR2 was significantly upregulated in HCC tissues and cell lines, and high KSR2 expression associated with poor prognosis in HCC patients. KSR2 knockdown significantly suppressed HCC cell proliferation, migration, and invasion in vitro and in vivo. Mechanistically, co-IP experiments identified that 14–3-3ζ complexed with KSR2, and elevated 14–3-3ζ increased KSR2 protein levels in HCC cells. Importantly, Kaplan–Meier survival analysis showed that patients with both high KSR2 and high 14–3-3ζ expression levels had the shortest survival times and poorest prognoses. Interestingly, HCC cells overexpressing both KSR2 and 14–3-3ζ, rather than either protein alone, showed hyperactivated MAPK signaling and resistance to sorafenib.

**Conclusions:**

Our results provide new insights into the pro-tumorigenic role of KSR2 and its regulation of the MAPK pathway in HCC. The KSR2–14–3-3ζ interaction may be a therapeutic target to enhance the sorafenib sensitivity of HCC.

**Supplementary Information:**

The online version contains supplementary material available at 10.1186/s40364-022-00361-9.

## Introduction

In 2020, liver cancer was the sixth most common cancer globally and the fourth leading cause of cancer deaths. There are approximately 906,000 new cases and 830,000 deaths annually [1]. Of primary liver cancer cases, 75%–85% are hepatocellular carcinoma (HCC) [1]. The natural course of HCC is largely asymptomatic, and HCC is typically diagnosed at an advanced stage in many patients [2]. Chronic infection with hepatitis B virus (HBV) or hepatitis C virus (HCV), aflatoxin-contaminated foodstuffs, heavy alcohol intake, obesity, smoking, and type 2 diabetes are the most common risk factors for HCC [3].

Overactivation of the Ras/Raf/MEK/ERK, which is a major signaling branch of the MAPK pathway involved in the growth and differentiation of human cells, has well-defined roles in tumorigenesis. For example, point mutations in each member of the cascade are known drivers of tumor formation (Ras and Raf mutations) or indicators of poor prognosis (MEK and ERK mutations) [4]. In this pathway, scaffold proteins facilitate kinase activation by colocalizing the core kinase components and impact the duration and amplitude of signaling by interacting with positive and negative regulators of the cascade [5, 6]. Thus, scaffold proteins are critical to MAPK signaling.

Two scaffold proteins, kinase suppressors of Ras proteins (KSR1 and KSR2), were originally identified from genetic screens in *Drosophila* and *C. elegans* and are known regulators of the MAPK cascade [7–9]. KSR1 plays an important role in regulating the transformation potential of oncogenic Ras, neuronal and adipocyte differentiation, and replication lifespan [10–13]. Additionally, KSR1 is associated with various signal regulators, including 14–3-3, C-TAK1 kinase, protein phosphatase 2A (PP2A), the E3 ubiquitin ligase IMP, and casein kinase 2 (CK2) [14–19]. A related protein, KSR2, can interact with several signaling components of the MAPK pathway, including Ras, CRAF, MEK1, and ERK1/2 [20]. Murine KSR2 shares 43% sequence identity (54% similarity) with murine KSR1 and possesses all of the conserved domains (CA1-CA5) previously identified in KSR family members, including residues that interact with the 14–3-3 proteins [9, 21]. However, KSR2 is unique in that it contains a 63-amino acid region located between the CA2 and CA3 domains. This domain interacts with AMP-activated protein kinase (AMPK), a phylogenetically conserved Ser/Thr protein kinase that acts as a fuel sensor, monitoring cellular energy status in eukaryotes [22]. Recent studies revealed the structure of KSR2-MEK1, suggesting how KSR2 interacts with BRAF to regulate MEK activation [23]. KSR2 interacts with a regulatory Raf molecule in cis to induce a conformational switch of MEK, facilitating MEK phosphorylation by a separate catalytic Raf molecule in trans [23]. Thus, KSR proteins appear to play critical roles in regulating multiple cell fates.

KSR1 associates with 14–3-3 proteins, members of a family of highly conserved cellular proteins that act via a pS/pT-binding motif to play essential roles in various cellular processes including signal transduction, cell cycle, apoptosis, cellular metabolism, stress responses, cytoskeleton organization, and malignant transformation [24, 25]. To date, seven mammalian 14–3-3 isoforms (β, γ, ε, ζ, η, θ/τ, σ) that interact with other critical cellular proteins during tumor development and progression have been identified [25, 26]; 14–3-3ζ is significantly overexpressed in HCC cells and tissues [16]. However, the role of the KSR2–14–3-3ζ association in hepatocarcinogenesis has not been investigated.

In this study, we found that KSR2 was upregulated in HCC and promoted tumor cell proliferation through the MAPK pathway. Small-sample clinical verification and preliminary functional experiments demonstrated the roles of KSR2 in HCC growth in vitro and in vivo. KSR2-interacting proteins were identified using co-IP and proteomic analyses, and their functions and effects on cell growth were further verified. Our findings reveal the underlying molecular mechanisms and demonstrate the potential clinical use of KSR2 in future HCC treatments.

## Materials and methods

### Cell lines, cell culture, and transfection of lentiviral vectors

The human HCC cell lines HuH7, PLC/PRF/5, MHCC97H, Hep3B, and HepG2 were obtained from the American Type Culture Collection (ATCC, Manassas, VA) and the cell library of the Chinese Academy of Sciences. All cell lines were cultured in the providers’ recommended medium supplemented with 10% fetal bovine serum (Gibco, South America origin), 100 μg/mL penicillin (Yeasen, Shanghai, China), and 100 μg/mL streptomycin (Yeasen, Shanghai, China) at 37 °C in a humidified 5% CO_2_-containing atmosphere. A KSR2 lentiviral vector was constructed (Genomeditech, Shanghai, China). Stable transfectants were characterized by quantitative real-time polymerase chain reaction (qPCR) or western blotting. Table S2 lists the targets of shKSR2.

### RNA extraction and real-time polymerase chain reaction assay

Total RNA was extracted from HCC cells and tissues using TRIzol Reagent (Sigma-Aldrich, USA) according to the manufacturer’s protocol. cDNA was synthesized by random primers and the HifairII 1st strand cDNA synthesis kit (Yeasen, Shanghai, China). Real-time polymerase chain reaction (qPCR) was performed using the Hieff qPCR SYBR green master mix (Yeasen, Shanghai, China). PCR conditions were: 95 °C for 30 s followed by 40 cycles of 95 °C for 10 s, and 60 °C for 30 s. Table S3 lists the PCR primers. *ACTB* was used as the internal control.

### Protein extraction and western blotting

Cell and tissue proteins were extracted using lysis buffer for WB/IP assays (Beyotime Institute of Biotechnology, China) with a phosphorylated protease inhibitor cocktail (Yeasen, Shanghai, China) and a proteinase inhibitor cocktail (Yeasen, Shanghai, China). Protein concentrations were determined using a BCA protein quantification kit (Yeasen, Shanghai, China). Proteins were separated by SDS-PAGE and transferred to polyvinylidene fluoride (PVDF) membranes (Millipore, USA). Membranes were incubated with primary antibodies overnight at 4 °C and probed with secondary antibodies at room temperature for 1–2 h. The following antibodies were used: anti-KSR2 (1:500, Abnova, Taiwan, China), anti-CRAF (1:1000, CST, Massachusetts, USA), anti-p-CRAF (1:1000, CST, Massachusetts, USA), anti-MEK1/2 (1:1000, CST, Massachusetts, USA), anti-p-MEK1/2 (1:1000, CST, Massachusetts, USA), anti-ERK1/2 (1:1000, CST, Massachusetts, USA), anti-p-ERK1/2 (1:1000, CST, Massachusetts, USA), anti-14–3-3ζ (1:1000, CST, Massachusetts, USA), anti-Cleaved-Caspase 3 (1:500, Arigo, Taiwan, China), and anti-β-actin (1:1000, CST, Massachusetts, USA).

### Cell transfections

Small interfering RNA (siRNA) oligonucleotides for 14–3-3ζ were designed and synthesized by Genomeditech (Shanghai, China). Table S4 shows the primer sequences for the siRNAs. Transient transfection was performed using the Lipofectamine 2000 reagent (Invitrogen, Carlsbad, USA) according to the manufacturer’s instructions. After transfection for 48 h, cells were used for functional assays, including cell viability assays, RNA extraction, and western blotting.

### Colony formation assays

For cell viability assays, 1000 cells were placed in a 6-well plate and maintained with 10% FBS-Dulbecco’s Modified Eagle’s Medium (DMEM) for 2 weeks. Colonies were fixed with methanol and stained with 0.1% crystal violet for 10 min. A clone including more than 50 cells was counted as positive.

### Wound healing migration

Cells were seeded into 6-well plates and grown to approximately 90% confluence. Cell monolayers were scratched with a 20-μL sterile pipette tip. Cells were rinsed with phosphate-buffered saline and cultured in DMEM supplemented with 1% fetal bovine serum. Cell migration was photographed 0 h and 48 h after scratching using an inverted microscope (Olympus, Tokyo, Japan).

### Invasion assays

Matrigel invasion assays were performed in 24-well plates with 8 μm-pore size chamber inserts (Corning, New York, USA). Upper chambers were precoated with 60 μL of Matrigel. After 30 min coating with Matrigel, 2 × 10^4^ cells in 200 μL of serum-free culture medium were seeded into each well of the upper chamber, and 500 μL of medium supplemented with 10% fetal bovine serum (FBS) were added to the lower chamber. Chambers were incubated at 37 °C with 5% CO_2_ for 72 h, and then cells that migrated through the membrane were fixed with 4% paraformaldehyde, stained with 0.1% crystal violet for 30 min, and imaged and counted under a light microscope (Olympus, Tokyo, Japan).

### In vivo tumor growth

All animal experiments were approved by the Animal Ethics Committee of Zhongshan Hospital affiliated with Fudan University. Experiments used 6-week-old male BALB/c-nu/nu mice raised by Zhongshan Hospital. Mice were housed and experiments performed in a specific pathogen-free (SPF) animal facility. For the in vivo xenograft assays, 5 × 10^6^ PLC/PRF/5 cells stably expressing shKSR2 or the negative control and 5 × 10^6^ PLC/PRF/5 cells stably expressing KSR2 or the lentiviral vector were individually subcutaneously inoculated into the dorsal right flanks of nude mice (6 per group). Size of the tumors was measured twice a week. Tumor volume was measured using calipers and calculated as V = (length × width^2^)/2. After 3–4 weeks, mice were euthanized and tumors were harvested and fixed in 10% neutral phosphate-buffered formalin. Fixed tumors were stained via immunohistochemistry. In another experiment, BALB/c-nu/nu mice were injected subcutaneously with PLC/PRF/5 cells (5 × 10^6^ cells/mouse) and treated with sorafenib (20 mg/kg gavage, once every other day) at day five for two weeks. On day 12 after the start of treatment, tumors were removed.

### Co-immunoprecipitation (co-IP) and protein mass spectrometry

Cells were cultured to > 90% confluence then lysed in immunoprecipitation lysis buffer (Beyotime Shanghai, China) with protease and protein phosphatase inhibitors. Lysates were incubated with 20 μL of protein A/G magnetic beads (MedChemExpress, New Jersey, USA) for 2 h. After incubating, the beads were removed, and 5–10 μL of primary antibody (KSR2) or isotype IgG was added to the supernatant and the samples mixed with gentle rocking at 4 °C overnight to capture the fusion proteins. Then, 20 μL of protein A/G beads was added and the immunoprecipitation mixes incubated for 2 h. Magnetic beads were collected by placing the tube in the appropriate magnetic separator. Beads were washed three times with cooled PBS buffer to remove nonspecifically bound proteins. Bound fusion proteins were eluted from the beads. Peptides were then analyzed (Bioprofile, Shanghai, China) with the Easy-nLC1200 chromatographic system (Thermo Fisher, Massachusetts, USA) and Q-exactive Plus mass spectrometer (Thermo Fisher, Massachusetts, USA). Protein identification was performed using MaxQuant1.6.1.0.

### Immunofluorescence (IF)

HCC tissue was fixed in 4% paraformaldehyde at 4 °C and then embedded in optimal cutting temperature compound and sectioned (20-μm thick) using a freezing microtome (Leica, Witzlar, Germany). After antigen retrieval, sections were blocked in phosphate-buffered saline supplemented with 5% bovine serum albumin for 1 h. Sections were then incubated with primary antibodies overnight at 4 °C. Secondary antibodies were Alexa Fluor 594-conjugated anti-rabbit IgG (1:200, Yeasen, Shanghai, China) or Alexa Fluor 488 anti-mouse IgG (1:200, Yeasen, Shanghai, China). Slides were exposed to DAPI nuclear stain (1:200, Yeasen, Shanghai, China) for 20 min, before being sealed with antifade mounting medium (Yeasen, Shanghai, China) and glass coverslips. Images were captured using a fluorescence microscope (Leica, Witzlar, Germany).

### Clinical specimens and tissue microarray (TMA)

A tissue microarray containing 198 paired HCC tissues and matched adjacent noncancerous tissues as well as fresh human hepatocellular carcinoma tissues and matched adjacent noncancerous tissues for qPCR and western blot analyses were collected from the Department of Liver Cancer at Zhongshan Hospital affiliated with Fudan University, Shanghai, China, from 2007 to 2020. All specimens were immediately snap-frozen in liquid nitrogen after surgical removal. Informed consent was obtained from all subjects to use the specimens described in this study. The procedure related to human subjects was approved by the Ethics Committee of the Fudan University.

### Immunohistochemistry (IHC)

Tissues were fixed in 4% paraformaldehyde, washed three times with PBS, transferred to 70% ethanol, and then embedded in paraffin and sectioned according to standard procedures. Sections were dewaxed with a graded ethanol series. After antigen retrieval, the tissues were stained using the Streptavidin Peroxidase IHC assay kit (ZSGB-Bio, Beijing, China). The primary antibodies were anti-KSR2 (1:100, Abnova, Taiwan, China), anti-14–3-3ζ (1:800, CST, Massachusetts, USA), anti-ERK1/2 (1:400, CST, Massachusetts, USA, anti-Cleaved-Caspase 3 (1:100, CST, Massachusetts, USA) and anti-KI67 (1:100, Abcam, Cambridge, UK). The average gray value of each image was used to quantify the expression level using Image-Pro Plus 6.0 software.

### Apoptosis analysis

Cell apoptosis was analyzed using the Annexin V-FITC/Propidium Iodide (PI) Apoptosis Detection Kit (Bio-platform, Shanghai, China) according to the manufacturer’s instructions. PLC/PRF/5 cells were stained with FITC and PI and then analyzed by fluorescence-activated cell sorting using the LSRFortessa (BD Biosciences, CA, USA). Cell apoptosis data were analyzed by the Flowjo V10.2 software.

### Statistical analysis

The statistical differences between groups were analyzed by χ^2^ analysis or two-tailed Student’s t tests. The Kaplan–Meier method was used for univariate survival analysis, and the log-rank test was used to assess the difference between survival curves. Spearman’s correlation coefficient was used to determine the correlation between the expression levels of KSR2 and 14–3-3ζ. Univariate and multivariate tests were carried out using two-sided Cox univariate analyses. Data are presented as the mean ± standard deviation (SD) or the mean ± standard error of the mean (SEM). Differences were considered statistically significant at *p* values < 0.05.

## Results

### Public expression databases show that KSR2 is upregulated in tumor tissues

**Fig. 1:**
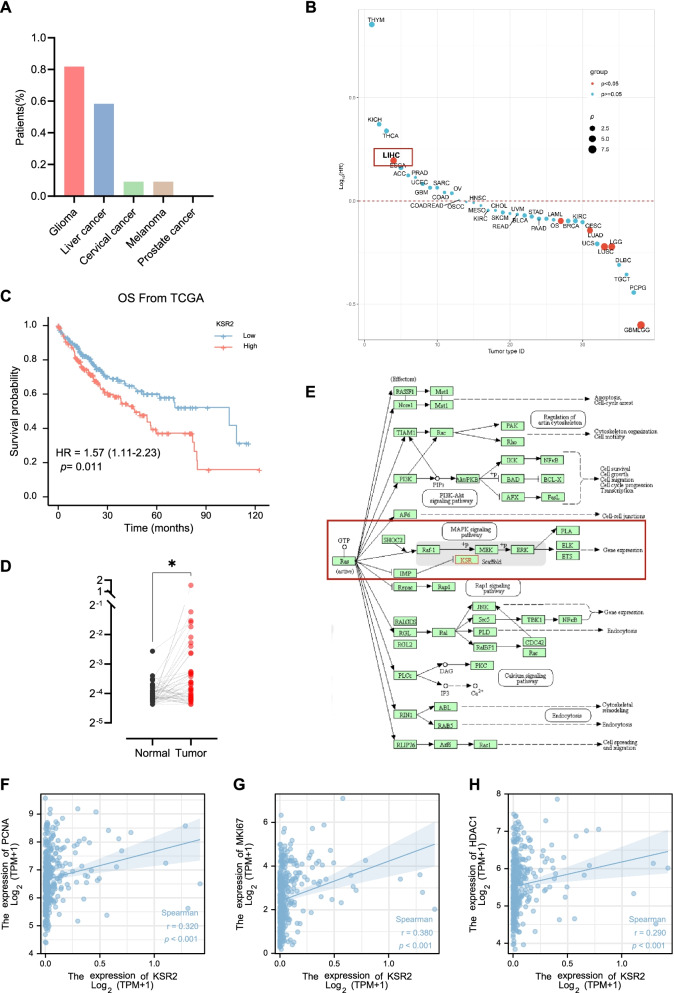
KSR2 is up-regulated in tumor tissues based on publicly available expression data. **A** The protein expression of KSR2 in various cancer tissues in the Human Protein Atlas database. **B** Bubble plot of survival curves for various malignant cancers. **C** Analysis of KSR2 expression and OS survival in liver cancer samples from The Cancer Genome Atlas (TCGA) database. **D** KSR2 transcription data in 50 pairs of cancer and adjacent tissues in the TCGA database. **E** Schematic diagram of MAPK signaling pathway derived from KEGG. **F** Expression level of proliferating cell nuclear antigen (PCNA) and KSR2 in HCC. **G** The expression level of human Ki67 (MKI67) and KSR2 in HCC. **H** The expression level of histone deacetylase 1 gene (HDAC1) and KSR2 in HCC. *p < 0.05; **p < 0.01; ***p < 0.001

Among five malignant tumor types, glioma and liver cancer had the highest expression of KSR2 based on data from the Human Protein Atlas (Fig. 1A). In these available data, high expression of KSR2 is the only meaningful risk factor (*p* < 0.05) for the occurrence of liver cancer (Fig. 1B). Moreover, data mining from The Cancer Genome Atlas (TCGA) showed that HCC patients with high KSR2 expression had low overall survival (OS) (Fig. 1C) and HCC tissues (T) had higher KSR2 levels than nontumor tissues (NT) (Fig. 1D). As shown in the schematic diagram of MAPK signaling pathway (Fig. 1E), KSR2 is an important scaffold in the MAPK pathway. To explore the relationship between KSR2 and tumor cell proliferation, we examined co-expression of KSR2 and the proliferating cell nuclear antigen (PCNA), human Ki67 (MKI67) protein, and histone deacetylase 1 (HDAC1). Expression of each of these proliferative markers (PCNA, MKI67, and HDAC1) positively correlated with that of KSR2, indicating KSR2 may promote tumor proliferation (Fig. 1F-H).

### KSR2 is significantly up-regulated in HCC and associates with a poor prognosis

**Fig. 2:**
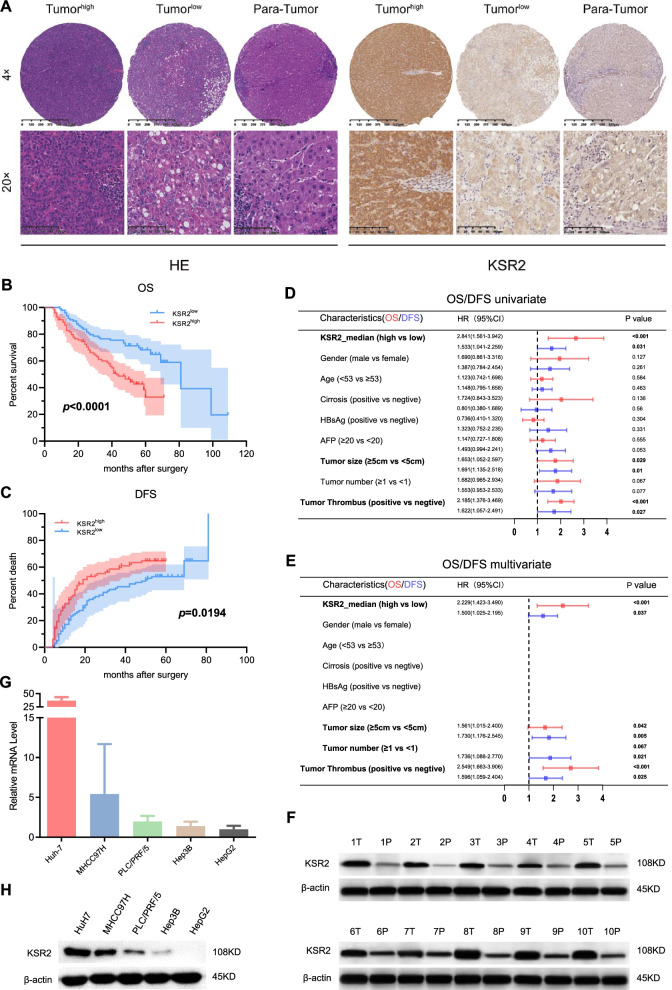
Elevated KRS2 is associated with poor prognosis. **A** The expression of KSR2 in cancer tissues in TMA, expression in adjacent normal tissues, and H and E staining. **B** The relationship between overall survival and expression of KSR2 was analyzed by Kaplan–Meier analysis. **C** The relation between disease-free survival and expression of KSR2 was analyzed by Kaplan–Meier analysis. **D**, **E** Univariate and multivariate cox analysis of OS and DFS in HCC patients. **F** Western blot analysis of KSR2 protein levels in 10 pairs of fresh liver cancer and adjacent tissues. **G** qPCR analysis of the mRNA levels of KSR2 in five liver cancer cell lines. **H** Western blot analysis of the protein levels of KSR2 in five liver cancer cell lines

To further verify that KSR2 promotes HCC, we examined expression of KSR2 in tissue microarrays that were created with tumor and adjacent normal liver tissues from 198 HCC patients who underwent radical resection from 2007 to 2017. KSR2 was highly expressed in tumors but low in adjacent tissues (Fig. 2A). We analyzed the correlation between KSR2 expression and clinicopathological indicators (Tab. 1). We further analyzed the relationship between KSR2 expression and the prognosis of patients with liver cancer and found that patients with high KSR2 expression had a shorter OS (*p *< 0.0001, Fig. 2B) and disease-free survival (DFS) (*p *= 0.0194, Fig. 2C) than patients with low KSR2 expression. Univariate analysis of OS revealed that associated factors were tumor size and tumor thrombus (*p* < 0.05, Fig. 2D); multivariate analysis identified tumor size and tumor thrombus as associated factors (*p* < 0.05, Fig. 2E). Single-factor analysis for DFS found that associated factors included tumor size and tumor thrombus (*p* < 0.05, Fig. 2D); multivariate analysis found tumor size, tumor number, and tumor thrombus (*p* < 0.05) to be associated factors (Fig. 2E). In addition, western blot analysis showed that KSR2 protein levels were significantly higher in HCC samples than in matched adjacent normal tissues (*n* = 10, Fig. 2F).

**Table 1:** Correlation between KSR2 levels in 198 HCC patients and their clinicopathologic characteristics

Variate	n	KSR2 expression		
		Low	High	*p* value
Age				0.200
< 53	103	47	56	
≥ 53	95	52	43	
Gender				1.000
Male	170	85	85	
Female	28	14	14	
HBsAg				0.852
No	35	17	18	
Yes	163	82	81	
Anti-HCV				0.902
No	7	4	3	
Yes	191	95	96	
Liver cirrhosis				0.817
No	21	11	10	
Yes	177	88	89	
AFP				0.769
< 20 ng/mL	74	36	38	
≥ 20 ng/mL	124	63	61	
ALT				0.540
≤ 50 U/L	136	70	66	
> 50 U/L	62	29	33	
GGT				1.000
≤ 60 U/L	112	56	56	
> 60 U/L	86	43	43	
Number of tumors				0.182
1	165	79	86	
> 1	33	20	13	
Tumor size				**0.032**
< 5 cm	111	63	48	
≥ 5 cm	87	36	51	
Tumor differentiation				0.752
good /middle	142	70	72	
poor	56	29	27	
Tumor capsule				0.670
No	95	49	46	
Yes	103	50	53	
Stage				0.641
I	139	71	68	
II	59	28	31	

To verify this, the mRNA and protein levels of KSR2 in liver cancer cells were analyzed by qPCR and western blot. We found that the mRNA and protein levels of KSR2 were moderately upregulated in 5 HCC cell lines (Fig. 2G and H). Taken together, these results indicate that KSR2 is frequently upregulated in HCC and a high level of KSR2 is associated with short OS and high recurrence rates in HCC patients.

### KSR2 enhances HCC cell proliferation, migration, and invasion

**Fig. 3:**
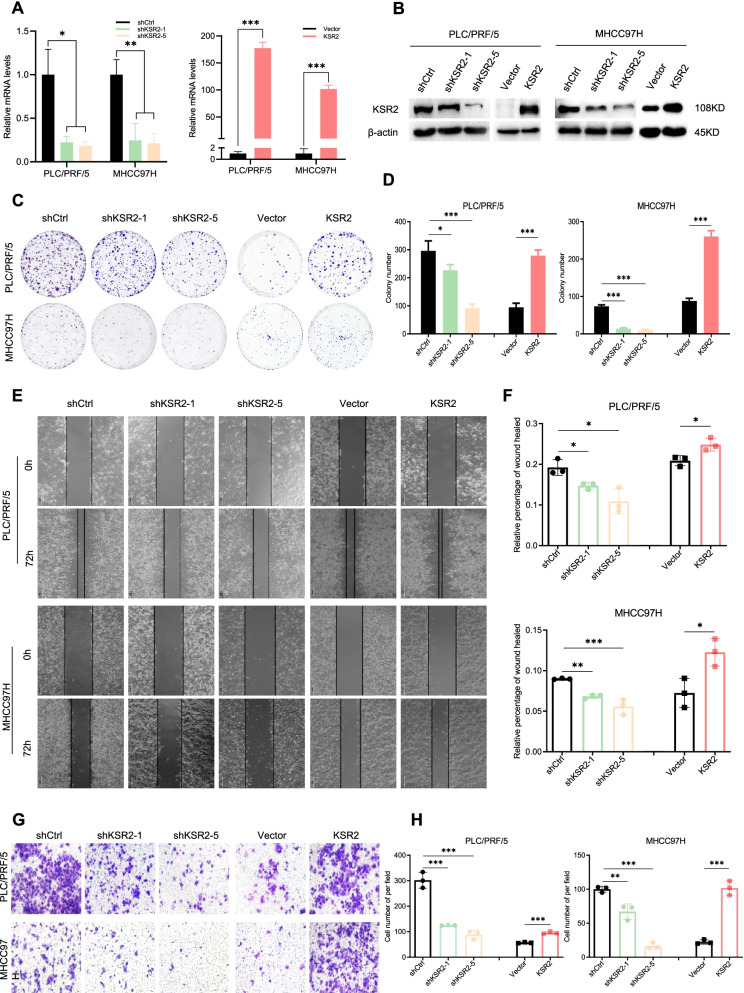
High level of KSR2 promotes HCC cell proliferation, migration, and invasion. **A** qPCR analysis of KSR2 knockdown and overexpression efficiency in PLC/PRF/5 and MHCC97H cells. **B** Western blot analysis of the knockdown and overexpression efficiency of KSR2 in PLC/PRF/5 cells. **C**, **D** Knockdown and overexpression of KSR2 affect the number of colonies formed by PLC/PRF/5 and MHCC97H cells. **E**, **F** Effect of knockdown and overexpression of KSR2 on the migration of PLC/PRF/5 and MHCC97H cells. **G**, **H** Cell invasion abilities of PLC/PRF/5 and MHCC97H cells evaluated by a matrigel invasion assay. *p < 0.05; **p < 0.01; ***p < 0.001

Of the tested HCC cell lines, MHCC97H and PLC/PRF/5 had intermediate levels of KSR2 expression. Therefore, we selected these lines for KSR2 knockdown and overexpression studies. Knockdown and overexpression efficacy was confirmed by qPCR and western blot (Fig. 3A and B). Five short hairpin RNAs were tested, and those demonstrating the most significant knockdowns (shRNA1 and shRNA5) were selected for subsequent experiments. Knockdown of KSR2 inhibited colony formation (Fig. 3C and D), cell migration (Fig. 3E and F), and cell invasion (Fig. 3G and H). In contrast, overexpression of KSR2 enhanced HCC cell proliferation, migration, and invasion in vitro (Fig. 3C-H).

### KSR2 promotes HCC growth in vivo

**Fig. 4:**
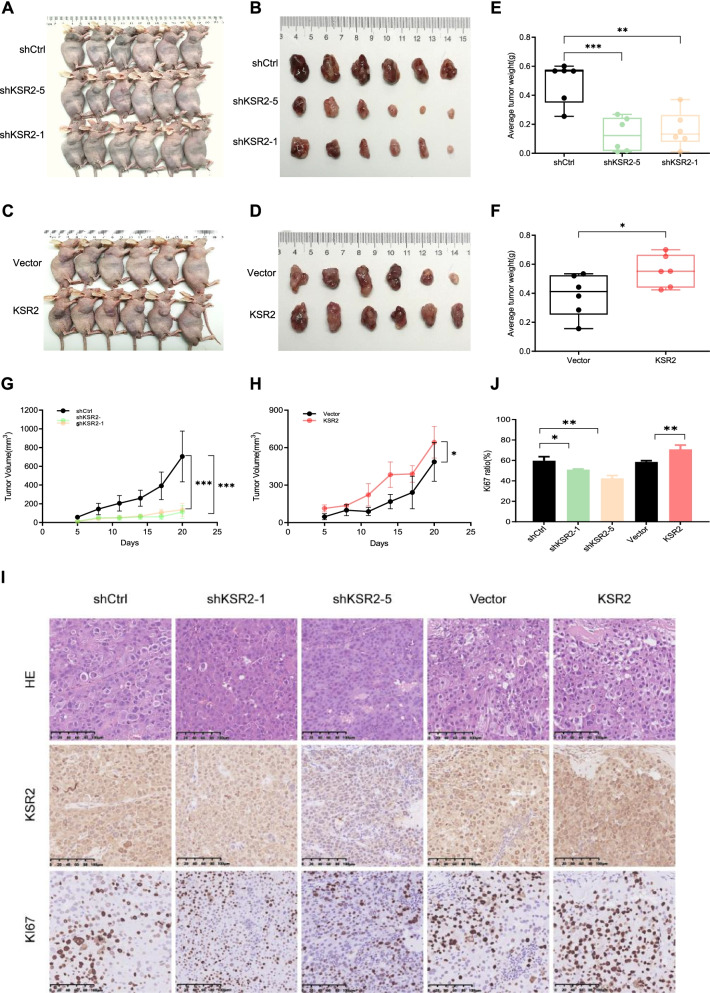
KSR2 promotes HCC growth in vivo. **A**, **B** KSR2 knockdown affects the ability of PLC/PRF/5 cells to form tumors in nude mice. **C**, **D** Overexpression of KSR2 affects the size of tumors derived from PLC/PRF/5 cells in nude mice. **E**, **F** Change in volume over time of subcutaneous tumors formed by cells with KRS2 overexpression and knockdown, relative to the control group. **G**, **H** Differences between the weight of the subcutaneous tumors formed after manipulating KSR2 levels and the weight of control tumors. **I** Immunohistochemical staining of subcutaneous tumor sections g KSR2 overexpression group, knockdown group, and control group. **J** The ratio of KI67 positive cells in the KSR2 overexpression group, knockdown group, and control group. *p < 0.05; **p < 0.01; ***p < 0.001

A xenograft model was used to determine whether KSR2 promotes HCC cell growth in vivo. Tumors derived from KSR2-knockdown cells were smaller and grew slower than those from the control cells, whereas overexpression of KSR2 increased tumor size and promoted tumor growth (Fig. 4A-H). IHC staining of the tissues resected from the xenograft tumors revealed that expression of the proliferation marker KI67 was increased in KSR2-overexpressing tumors but decreased in the KSR2-knockdown tumors (Fig. 4I and J). These results suggest that KSR2 promotes HCC growth in vivo.

### KSR2 serves as a promising target for sorafenib

**Fig. 5:**
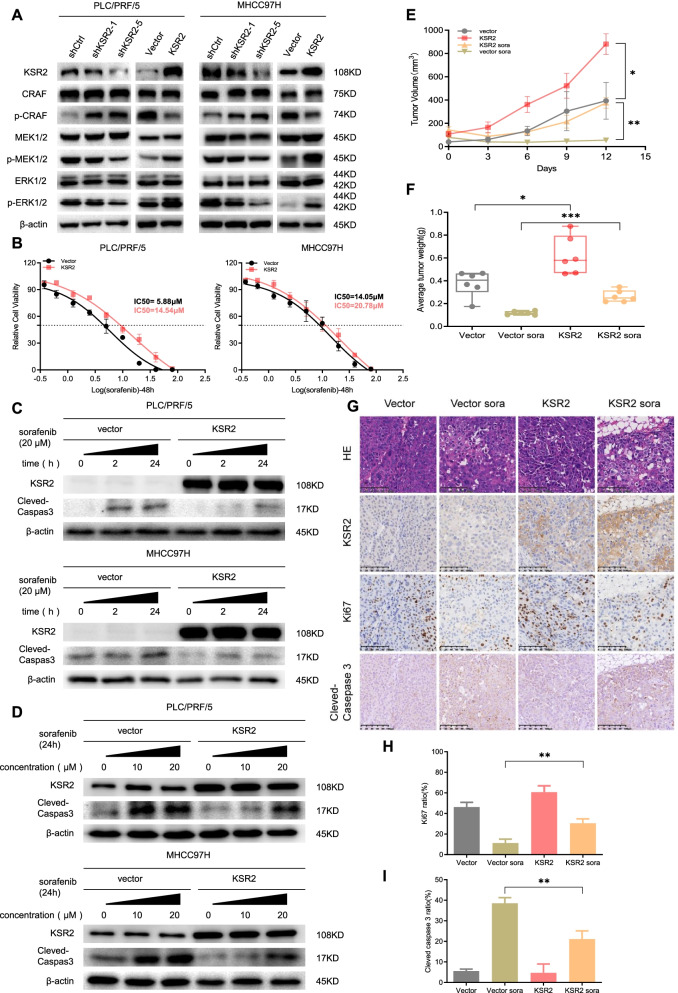
KSR2 serves as a promising target for sorafenib. **A** Western blot results show the effects of knockdown and overexpression of KSR2 on the expression of MAPK pathway markers p-cRaf, p-MEK1/2, and p-ERK1/2. **B** IC50 of sorafenib in PLC/PRF/5 and MHCC97H cells at 48 h of treatment. **C** Protein expression of Cleaved-Caspase3 in PLC/PRF/5 and MHCC97H cells exposed to sorafenib (20 μM) for different durations (0h, 2h, 24h). **D** Protein expression of Cleaved-Caspase3 in PLC/PRF/5 and MHCC97H cells treated with different concentrations of sorafenib (0 μM, 10 μM, 20 μM) for 24 h. **E** Time course showing the effect of sorafenib on the volume of subcutaneous tumors in KSR2-overexpression and control groups. **F** Effect of sorafenib on the weight of subcutaneous tumors from the KSR2-overexpression cells and the control groups. **G** Immunohistochemical staining of subcutaneous tumor sections from the KSR2 overexpression group and control groups following treatment with sorafenib. **H** Quantification of the number of KI67^+^ cells in the KSR2 overexpression and control groups following treatment with sorafenib. **I** Quantification of the number of Cleved caspase 3 area in the KSR2 overexpression group and control group following treatment with sorafenib. *p < 0.05; **p < 0.01; ***p < 0.001. *Abbreviation*: *sora* sorafenib

To elucidate the molecular mechanism underlying KSR2-induced HCC cell growth, we tested whether KSR2 affects signaling via the MAPK pathway in HCC cells. As shown in Fig. 5A, silencing of KSR2 in MHCC97H and PLC/PRF/5 cells arrested ERK1/2 phosphorylation. In contrast, KSR2, p-MEK1/2, and p-ERK1/2 were all elevated in MHCC97H and PLC/PRF/5 cells engineered to overexpress KSR2. These results indicate that KSR2 contributes to MAPK-induced HCC proliferation.

Considering that KSR2 appears to act via the MAPK pathway, we tested the effects of KSR2 overexpression on tumor-cell sensitivity to sorafenib, a drug that blocks tumor growth and angiogenesis by targeting both the MAPK pathway and receptor tyrosine kinases. Sorafenib was the first FDA-approved molecular-targeted drug used in the treatment of advanced HCC patients [27]. We found that KSR2 overexpression reduced the inhibitory effects of sorafenib on MHCC97H and PLC/PRF/5 cells (Fig. 5B). Figure 5C shows the time-course of apoptosis induced by sorafenib in MHCC97H and PLC/PRF/5 cells. KSR2 overexpression dramatically reduced apoptosis in these cells. Further, following 24 h of treatment, sorafenib induces apoptosis in a dose-dependent manner in MHCC97H and PLC/PRF/5 cells, but KSR2-overexpression reduces this effect (Fig. 5D).

KSR2 overexpression also abrogated the effect of sorafenib treatment in our tumor-xenograft model. KSR2 overexpression increased the tumor growth rate and KI67-positive cell numbers in both dimethyl sulphoxide (DMSO) and sorafenib-treated nude mice xenografts (Fig. 5E-H). Yet, Cleved-Caspase 3 presented a 17% reduction in positive area in sorafenib-treated overexpression (Fig. 5G and I), indicating it bypasses the growth suppressive effects of sorafenib.

### KSR2 interacts with 14–3-3ζ and promotes the proliferation of HCC cells through the MAPK pathway

**Fig. 6:**
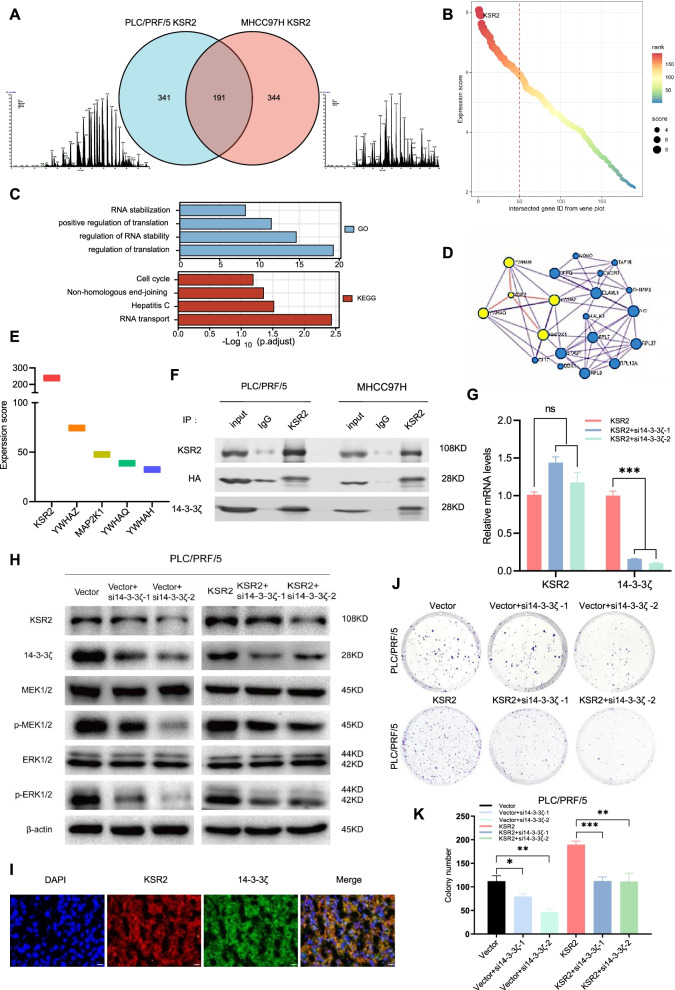
KSR2 interacts with 14–3-3ζ and promotes the proliferation of HCC cells through the MAPK pathway. **A** Protein interaction mass spectrum analyzed by immunoprecipitation in PLC/PRF/5 and MHCC97H cells. Venn diagram shows the intersection of the interacting proteins obtained after KSR2 overexpression in PLC/PRF/5 and MHCC97H cells. **B** Expression score of proteins interacting with KSR2. **C** GO and KEGG analysis of the Top 100 genes. **D** A protein–protein interaction (PPI) network of KSR2 with the hub genes. **E** Expression score of the hub genes interacting with KSR2. **F** Interaction between KSR2 and 14–3-3ζ was detected by co-IP in PLC/PRF/5 and MHCC97H cells. **G** qPCR confirming the transcription level of KSR2 after knocking down 14–3-3ζ as well as the knockdown efficiency of 14–3-3ζ. **H** Western blot showing the effects of knocking down 14–3-3ζ on phosphorylation in the KSR2 overexpression and control groups. **I **IF showing the localization of KSR2 and 14–3-3ζ in HCC tissue. **J** Clonogenic survival of control and KSR2-overexpressing but with 14–3-3ζ-knockdown cells. **K** Overexpression of KSR2 and knockdown of 14–3-3ζ affects the number of clones formed from PLC/PRF/5 cells. *p < 0.05; **p < 0.01; ***p < 0.001

To further explore how KSR2 promotes HCC, we sought to identify KSR2-interacting proteins. We performed KSR2-affinity purification from KSR2 overexpressing PLC/PRF/5 and MHCC97H cells followed by mass spectrometry and identified 191 potential KSR2 interacting proteins present in both cell lines (Fig. 6A). The 191 identified proteins are shown according to their expression score (Fig. 6B); the top-100 candidates were then analyzed by GO enrichment and KEGG pathway analysis (Fig. 6C, Tab. S5). We found these genes corresponding to proteins were enriched in the functions of “RNA translation and stabilization.” Application of MCODE algorithm to the top-100 genes was used to identify their neighborhoods where proteins are densely connected, revealing a 20-gene MCODE network including *KSR2* [28]. Interestingly, in the 4 hub genes directly linked to *KSR2*, 3 of them (*YWHAZ, YWHAQ, YWHZH*) belong to the 14–3-3 protein family that can facilitate protein folding and accumulation (Fig. 6D), and *YWHAZ* (coding 14-3-3ζ) scored the highest of the 4 hub genes (Fig. 6E). Therefore, we further explored the function of 14–3-3ζ protein.

Dougherty et al. reported that the 14–3-3 family plays an important role in regulating the intracellular localization of the KSR1 scaffold [21]. We speculated that 14–3-3ζ could also interact with KSR2 and promote activation of the MAPK pathway. Having found that 14–3-3ζ interacted with KSR2 in cells overexpressing KSR2, we confirmed this interaction by co-IP experiments (Fig. 6F). To explore the interaction mechanism between 14–3-3 and KSR2, we examined the mRNA levels of KSR2 before and after 14–3-3 protein knockdown. qPCR proved that knocking down 14–3-3 did not affect KSR2 mRNA levels (Fig. 6G), but reduced the protein level of KSR2 (Fig. 6H), so we speculated that 14–3-3ζ might act as a molecular chaperone to help KSR2 perform its function. Consistent with this, we demonstrated the interaction between the two proteins by IF of human HCC tissues, revealing that the KSR2 protein colocalizes with 14–3-3ζ in the cytoplasm and nucleus (Fig. 6I).

We examined the functional interaction of these two proteins in HCC cell lines by combined overexpression and knockdown experiments. KSR2 overexpression promoted HCC cell growth. However, when combined with knockdown of 14–3-3ζ, the proliferation and colony formation ability of KSR2-overexpressing and control cells decreased (Fig. 6J and K) accompanied by a decrease in the phosphorylation of MEK1/2 and ERK1/2 detected by western blotting (Fig. 6H). 14–3-3ζ knockdown rescue the effect of KSR2 high-expression on MEK1/2 and ERK1/2 phosphorylation. But 14–3-3ζ knockdown had a more dramatic effect on MEK1/2 and ERK1/2 phosphorylation in cells without KSR2 overexpression, indicating that KSR2 overexpression is high enough to mask the regulating effect of 14–3-3ζ. For this reason, we conclude that 14–3-3ζ is involved in the regulation of MAPK through effects on the expression of KSR2. These data collectively suggest that KSR2 may specifically interact with 14–3-3ζ to contribute to HCC cell growth.

### The “KSR2–14–3-3ζ effect” is related to poor HCC prognosis and decreased sensitivity to sorafenib

**Fig. 7:**
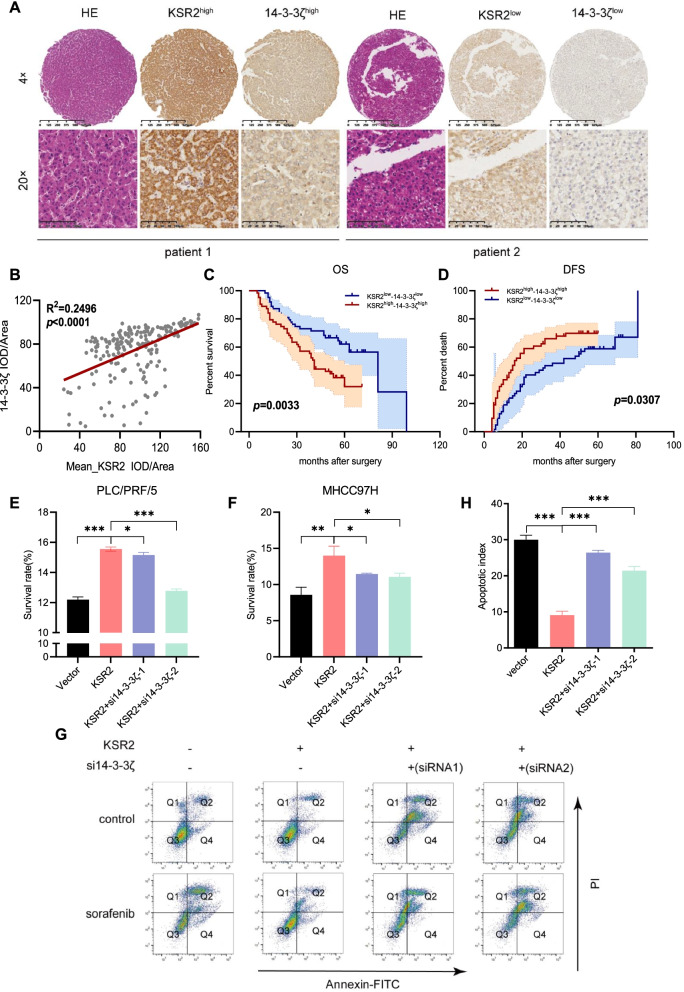
Combined overexpression of KSR2 and 14–3-3ζ is associated with poor prognosis and decreased HCC sensitivity to sorafenib. **A** Expression of KSR2 and 14–3-3ζ together in a TMA of cancer tissues and normal adjacent tissues, and H and E staining. **B** Relationship between the expression level of KSR2 and the expression level of 14–3-3ζ in a TMA. **C** Relationship between overall survival and expression of KSR2 and 14–3-3ζ assessed analyzed by Kaplan–Meier analysis. **D** Relationship between disease-free survival and expression of KSR2 and 14–3-3ζ assessed by Kaplan–Meier analysis. **E**, **F** Percentage of surviving PLC/PRF/5 cells and MHCC97H cells overexpressing KSR2 in the presence of sorafenib. *n* = 3 independent experiments. **G** Apoptosis in KSR2-overexpressing cells with 14–3-3ζ-knockdown after sorafenib treatment. **H** Percentage of apoptotic PLC/PRF/5 cells overexpressing KSR2 in the presence of sorafenib. *n* = 3 independent experiments. *p < 0.05; **p < 0.01; ***p < 0.001

To test whether the expression of KSR2 and 14–3-3ζ in HCC relates to the prognosis of patients, we performed IHC analysis of our TMA. Statistically, the overall expression of KSR2 and 14–3-3ζ was much higher in cancerous tissues than in the adjacent noncancerous tissues (Fig. 7A, Fig. S1A). In HCC tissues, 65.66% (130/198) expressed high level of 14–3-3ζ, but 14–3-3ζ expression revealed no impact on OS (*p* = 0.2662, Fig. S1B) and DFS (*p* = 0.3012, Fig. S1C). Importantly, KSR2 protein expression positively correlated with that of 14–3-3ζ, suggesting a potential “KSR2-14–3-3ζ effect” in HCC tissues (*R*^*2*^ = 0.2496, *p* < 0.0001, Fig. 7B). Kaplan–Meier analysis showed that patients with high expression of KSR2 and 14–3-3ζ had lower OS (*p* = 0.0033) and shorter DFS (*p* = 0.0307) compared with patients with concordant low expression of the two molecules (Fig. 7C and D).

**Fig. 8:**
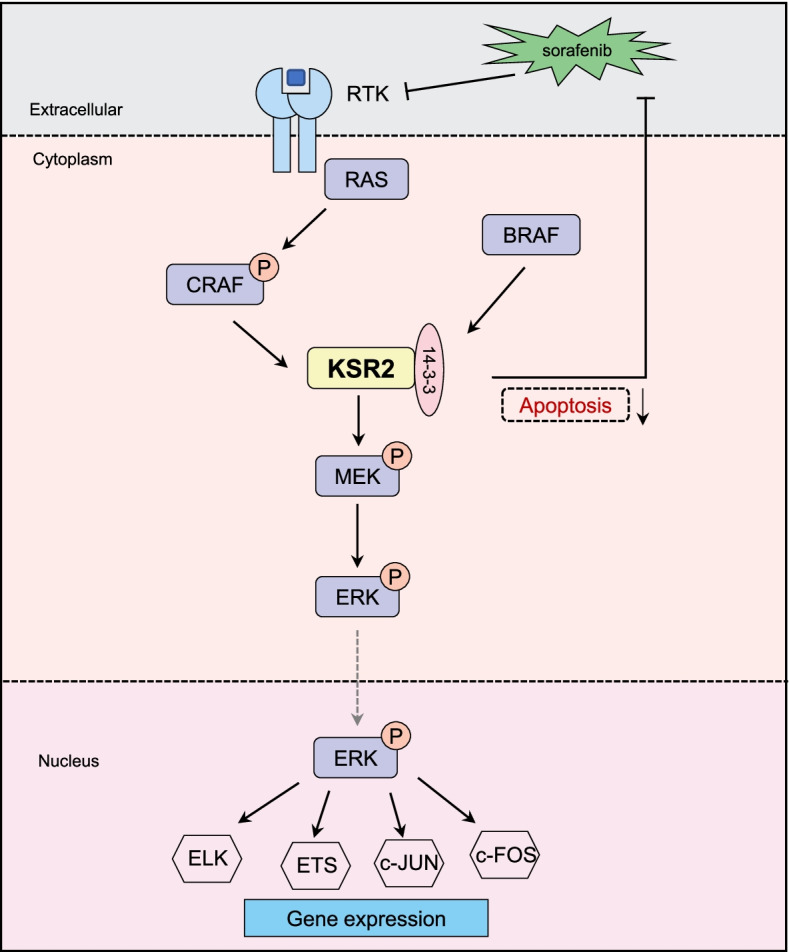
Schematic illustrating the mechanism by which KSR2 in regulates liver cancer growth and progression. In this proposed model, KSR2 can interact with 14–3-3ζ and form a complex, activating the Ras–Raf–MEK–ERK signaling pathway and thereby leading to the proliferation and growth of HCC cells

We examined the impact of 14–3-3ζ on sorafenib sensitivity of KSR2-overexpressing PLC/PRF/5 and MHCC97H cells. KSR2-overexpressing cells treated with sorafenib (20 μg/mL, 48 h, according to Fig. 5B) had a higher survival rate than control cells (20 μg/mL, 48 h), but reducing 14–3-3ζ levels enhanced HCC cell sensitivity to sorafenib (Fig. 7E-H). This indicates that 14–3-3ζ plays an important role in regulating the KSR2 scaffold. Thus, sorafenib in combination with a 14–3-3 inhibitor may be an effective treatment for KSR2-elevated HCC tumors (Fig. 8).

## Discussion

In the present study, we report the novel observation that the molecular scaffold KSR2 is a regulator of MAPK activity, and its overexpression promotes HCC development. Moreover, we showed that KSR2 associates with 14–3-3ζ, and 14–3-3ζ can upregulate the KSR2 protein in HCC cells. Importantly, both elevated KSR2 and 14–3-3ζ expression conferred sorafenib-resistance to HCC cells. Clinically, patients with high levels of both KSR2 and 14–3-3ζ expression had the shortest OS and highest recurrence rates. Thus, our findings reveal a novel pathway where KSR2 promotes tumorigenesis through MAPK signaling and provide a novel therapeutic target for treating HCC.

KSR1 and KSR2 have different but overlapping roles in cells. Both proteins can serve as a scaffold to promote ERK signaling, but KSR2 may be even more effective than KSR1, as growth factor-induced ERK activation in MEFs required nine times more KSR1 than KSR2 [29]. The enhanced activity of KRS2 may be arise from 1) KSR2 transferring to the plasma membrane and assembling the kinase cascade in response to growth factor stimulation more efficiently than KSR1 [16, 21, 30]; 2) the KSR2 scaffold complex being less affected by ERK-dependent feedback loops that negatively regulate KSR1-scaffolded complexes [30]; 3) KSR2 being more efficient than KSR1 in its ability to process MEK-dependent ERK phosphorylation. However, disrupting the interaction of KSR2 with ERK did not alter the biological action of KSR2 demonstrating that KSR2-dependent ERK activation is dispensable for KSR2-dependent cell proliferation. Thus, despite their strong homology and ability to activate the same kinase cascade, KSR2 and KSR1 affect cell proliferation differently as well as KSR2 being potentially more potent than KSR1 in promulgating transmission of signals from Raf to MEK and from MEK to ERK [11, 12, 31].

KSR1 and KSR2 also have unique but overlapping roles in mediating Ras signaling during *C. elegans* development [20]. KSR2 uniquely contributes to meiosis in *C. elegans* and is speculated to function by controlling ERK phosphorylation in oocytes, because the destruction of KSR2 leads to the acute loss of MPK1 activation. Recent work revealed that KSR2 receives a stimulatory signal from an activated Raf molecule and transmits this to regulate the activation of MEK fragments to catalyze the phosphorylation of Raf in human cells [23]. Our results further implicate KSR2 in playing an important role in the MAPK pathway.

The role of KSR2 in cancer has not been extensively explored. A study by Pooja Popli et al. [32] did report that splicing factor SF3B1 is involved in the maturation of KSR2 pre-mRNA to a mature RNA and that KSR2 acts downstream of SF3B1 function in endometrial cancers. An additional study reported KSR2 facilitates the activation of AMPK, which is essential for nutrient metabolism, maximum glycolysis, and oxidative phosphorylation (OXPHOS) capacity of neuroblastoma cells [22]. A new study identified several mutations in *KSR2* that are linked to the development of early-onset obesity and severe insulin resistance [33]. Further, deletion of *Ksr2* leads to obesity in mice, which suggests that it has a role in energy homeostasis [22]. These results raises the intriguing possibility that KSR2 serves not only as a scaffold, but also as a component of an energy and nutrient sensor that couples information about the nutritional environment and intracellular energy status of a cell to a kinase cascade with potent effects on cancer cell proliferation, differentiation, and survival.

KSR proteins also associate with members of the 14–3-3 protein family, a family that is ubiquitous in eukaryotic cells. 14–3-3 proteins regulate various important physiological activities of cells by binding to a wide range of protein ligands, like KSR proteins, they participate in MAPK signaling [26]. Recent studies describe autoinhibited and active-state structures of full-length BRAF in complexes with MEK1 and a 14–3-3 dimer (the ε and ζ isoforms) determined by cryo-electron microscopy [34]. In the inhibited state, the 14–3-3 protein dimer wraps the complex of BRAF and MEK; in the activated state, the 14–3-3 protein rearranges to help BRAF form an active back-to-back dimer. The 14–3-3 protein has a similar role in interacting with KSR family proteins, which have a similar structure to Raf. In the presence of activated Ras, KSR1 contains three additional sites of phosphorylation (Thr260, Thr274, and Ser443), all of which match the consensus motif (Px[S/T]P) for phosphorylation by MAPK [14]. KSR1 consistently showed a marked preference for binding to 14–3-3γ, and it was able to interact with some but not all 14–3-3 isoforms (η > β, ζ > τ) [35]. We showed that KSR2 interacts with 14–3-3ζ, an interaction whose function has not previously been investigated. Our data do, however, indicate that 14–3-3ζ contributes to the regulation of KSR2 in HCC.

There is increasing evidence indicating that some 14–3-3 isoforms are overexpressed in tumors and that 14–3-3 proteins are implicated in regulating tumor progression of various types of human malignancies [36]. During tumor formation and progression, 14–3-3ζ may be involved in the malignant transformation, proliferation, growth, and metastasis of tumor cells [37]. 14–3-3ζ levels may also modulate cancer cell sensitivity to treatment. For example, overexpression of miR-451 increased breast cancer cell resistance to paclitaxel mainly through downregulating 14–3-3ζ expression in vitro and in vivo [38]. Curiously, we found that reducing 14–3-3ζ levels enhanced the sensitivity of KSR2-overexpressing HCC cells to sorafenib.

Sorafenib, as the first-generation targeted drug, has proven beneficial to patients with advanced liver cancer. Sorafenib inhibits tumor cell proliferation, increases the rate of apoptosis, and inhibits tumor angiogenesis by inhibiting the serine–threonine kinases CRAF and BRAF and the receptor tyrosine kinase activity of vascular endothelial growth factor receptors (VEGFRs) and platelet-derived growth factor receptor β (PDGFR-β) [39, 40]. However, most patients develop resistance to sorafenib in the early stages. Acquired resistance is always established during long-term sorafenib exposure, whereby sorafenib itself acts as a selective force that favors the outgrowth of drug-resistant subclones. On this basis, oncoproteins like phosphorylated ERK1/2 might provide promising targets for sorafenib response [41]. Our study shows that KSR2 activates phosphorylation of ERK1/2 and reduces the sensitivity of HCC cells to sorafenib, indicating that KSR2 may be a promising HCC biomarker and a potential target for inhibiting the progression of HCC.

## Conclusions

In summary, the present study demonstrates that KSR2 regulates the proliferation and growth of hepatocellular carcinoma. Further, KSR2 and 14–3-3ζ interact and activate the MAPK signaling pathway, which ultimately increases the proliferation of HCC cells (Fig. 8). More importantly, our investigation reveals that high expression levels of KSR2 and 14–3-3ζ are significantly correlated with tumor growth and serve as independent prognostic factors for the poor outcomes of HCC patients. Additionally, KSR2 inhibition enhances HCC cell sensitivity to sorafenib. Altogether, KSR2 might be a promising HCC biomarker and potential target to suppress HCC progression.

## Supplementary Information

**Additional file 1.**

**Additional file 2.**

## Data Availability

Available upon request.

## Abbreviations

KSR1: Kinase suppressors of Ras 1
KSR2: Kinase suppressors of Ras 2
HCC: Hepatocellular carcinoma
IHC: Immunohistochemistry
co-IP: Co-immunoprecipitation
OS: Overall survival
DFS: Disease-free survival
