# Supplementary material for: KSR2-14–3-3ζ complex serves as a biomarker and potential therapeutic target in sorafenib-resistant hepatocellular carcinoma
Source: Biomark Res. 2022 Apr 25;10:25. doi: 10.1186/s40364-022-00361-9 (PMC9036720; doi:10.1186/s40364-022-00361-9)
Supplement: Supplementary file 1 — Additional file 1. [file 40364_2022_361_MOESM1_ESM.docx]

**Supplementary table 1.** 10 HCC patients and their clinicopathologic characteristics in figure 2F

| No. | Age | Gender | HCC | Liver cirrhosis | HBV infection | AFP  (ng/mL) | Tumor size (cm) | Tumor capsule |
| --- | --- | --- | --- | --- | --- | --- | --- | --- |
| 1 | 74 | Male | Yes | Yes | Yes | 2.5 | 7.0 | Yes |
| 2 | 35 | Male | Yes | Yes | Yes | 198 | 4.0 | Yes |
| 3 | 67 | Female | Yes | Yes | Yes | 2 | 5.0 | No |
| 4 | 73 | Male | Yes | Yes | Yes | 7030 | 4.0 | No |
| 5 | 55 | Male | Yes | Yes | Yes | 5 | 6.5 | No |
| 6 | 71 | Female | Yes | Yes | Yes | 60500 | 8.0 | Yes |
| 7 | 64 | Male | Yes | Yes | Yes | 6000 | 6.0 | Yes |
| 8 | 53 | Male | Yes | Yes | Yes | 4725 | 7.0 | Yes |
| 9 | 60 | Male | Yes | Yes | Yes | 5 | 3.0 | Yes |
| 10 | 46 | Male | Yes | Yes | Yes | 2.4 | 7.0 | No |

**Supplementary table 2.** The shRNA sequence in the article

| Primer name | Primer sequence |
| --- | --- |
| Primer-T1 | CCGGGCATCCCACTACTACAAATACCTCGAGGTATTTGTAGTAGTGGGATGCTTTTT |
| Primer-B1 | AATTAAAAAGCATCCCACTACTACAAATACCTCGAGGTATTTGTAGTAGTGGGATGC |
| Primer-T2 | CCGGGCAAGCTGGTGAAGTACTTCACTCGAGTGAAGTACTTCACCAGCTTGCTTTTT |
| Primer-B2 | AATTAAAAAGCAAGCTGGTGAAGTACTTCACTCGAGTGAAGTACTTCACCAGCTTGC |
| Primer-T3 | CCGGGGAAGGAAATCCATTACTTCACTCGAGTGAAGTAATGGATTTCCTTCCTTTTT |
| Primer-B3 | AATTAAAAAGGAAGGAAATCCATTACTTCACTCGAGTGAAGTAATGGATTTCCTTCC |
| Primer-T4 | CCGGGGAGCAAATCCCACGAGTTCCCTCGAGGGAACTCGTGGGATTTGCTCCTTTTT |
| Primer-B4 | AATTAAAAAGGAGCAAATCCCACGAGTTCCCTCGAGGGAACTCGTGGGATTTGCTCC |
| Primer-T5 | CCGGGCCTTGAACCTCAAGATCCACTCGAGTGGATCTTGAGGTTCAAGGTTTTT |
| Primer-B5 | AATTAAAAACCTTGAACCTCAAGATCCACTCGAGTGGATCTTGAGGTTCAAGGC |
| Primer-NC-T | CCGGGTTCTCCGAACGTGTCACGTCTCGAGACGTGACACGTTCGGAGAATTTTT |
| Primer-NC-B | AATTAAAAATTCTCCGAACGTGTCACGTCTCGAGACGTGACACGTTCGGAGAAC |

**Supplementary Table 3.** The primers used in this article

| Primer name | Primer sequence |
| --- | --- |
| KSR2-F | CTGCATCGGAGCAAATCCC |
| KSR2-R | CTGCTGTGGATCTTGAGGTTC |
| 14-3-3ζ-F | CTCTCTTGCAAAGACAGCTTTT |
| 14-3-3ζ-R | GTCCACAATGTCAAGTTGTCTC |

**Supplementary table 4.** The siRNA sequence in the article

| siRNA name | sequence |
| --- | --- |
| 14-3-3ζ-NC | UUCUCCGAACGUGUCACGU tt  ACGUGACACGUUCGGAGAA tt |
| 14-3-3ζ-si-1 | GCAGAGAGCAAAGUCUUCUAU tt |
|  | AUAGAAGACUUUGCUCUCUGC tt |
| 14-3-3ζ-si-2 | GCUCGAGAAUACAGAGAGAAA tt |
|  | UUUCUCUCUGUAUUCUCGAGC tt |
| 14-3-3ζ-si-3 | GCAAUUACUGAGAGACAACUU tt |
|  | AAGUUGUCUCUCAGUAAUUGC tt |

**Supplementary table 5.** The top 100 protein of co-IP

| Protein names | Gene names | Score |
| --- | --- | --- |
| Insulin-like growth factor 2 mRNA-binding protein 3 | IGF2BP3 | 276.785 |
| Myosin-9 | MYH9 | 238.82 |
| tRNA-splicing ligase RtcB homolog | RTCB | 200.48 |
| A-kinase anchor protein 8-like | AKAP8L | 192.08 |
| Kinase suppressor of Ras 2 | KSR2 | 191.9025 |
| Ubiquitin-associated protein 2-like | UBAP2L | 166.13 |
| ATP-dependent RNA helicase DDX54 | DDX54 | 163.4003 |
| Ataxin-2-like protein | ATXN2L | 158.51 |
| ATP-dependent RNA helicase DDX1 | DDX1 | 157.4415 |
| Regulator of nonsense transcripts 1 | UPF1 | 150.29805 |
| Kinesin-like protein;Centromere-associated protein E | CENPE | 147.73762 |
| Clathrin heavy chain;Clathrin heavy chain 1 | CLTC | 147.01775 |
| Interleukin enhancer-binding factor 3 | ILF3 | 139.225 |
| Heterogeneous nuclear ribonucleoprotein Q | SYNCRIP | 122.5135 |
| Plakophilin-2 | PKP2 | 118.0125 |
| X-ray repair cross-complementing protein 6 | XRCC6 | 115.5615 |
| Nuclear fragile X mental retardation-interacting protein 2 | NUFIP2 | 112.19915 |
| X-ray repair cross-complementing protein 5 | XRCC5 | 110.15237 |
| Spermatogenesis-associated serine-rich protein 2 | SPATS2 | 109.7795 |
| RNA-binding motif protein, X chromosome;RNA-binding motif protein, X chromosome, N-terminally processed;RNA binding motif protein, X-linked-like-1 | RBMX;RBMXL1 | 109.2625 |
| 14-3-3 protein gamma;14-3-3 protein gamma, N-terminally processed | YWHAG | 103.742 |
| Fragile X mental retardation syndrome-related protein 2 | FXR2 | 100.873085 |
| Myosin regulatory light chain 12A;Myosin regulatory light chain 12B;Myosin regulatory light polypeptide 9 | MYL12A;MYL12B;MYL9 | 98.98005 |
| 40S ribosomal protein S29 | RPS29 | 94.4015 |
| Apoptosis-inducing factor 1, mitochondrial | AIFM1 | 91.145 |
| RNA-binding protein 14 | RBM14 | 89.86135 |
| Translocon-associated protein subunit gamma | SSR3 | 85.78735 |
| Zinc finger protein 609 | ZNF609 | 82.392075 |
| LINE-1 retrotransposable element ORF1 protein | L1RE1 | 77.892 |
| 60 kDa heat shock protein, mitochondrial | HSPD1 | 77.042 |
| Insulin-like growth factor 2 mRNA-binding protein 1 | IGF2BP1 | 75.31275 |
| Putative helicase MOV-10 | MOV10 | 75.24385 |
| Nuclease-sensitive element-binding protein 1 | YBX1 | 73.453 |
| UPF0568 protein C14orf166 | C14orf166 | 71.92 |
| Actin-binding protein anillin | ANLN | 71.5655 |
| LIM and SH3 domain protein 1 | LASP1 | 71.0185 |
| TATA-binding protein-associated factor 2N | TAF15 | 69.7529 |
| DBIRD complex subunit ZNF326 | ZNF326 | 69.4095 |
| Constitutive coactivator of PPAR-gamma-like protein 1 | FAM120A | 68.973 |
| Phosphate carrier protein, mitochondrial | SLC25A3 | 68.7795 |
| Double-stranded RNA-binding protein Staufen homolog 1 | STAU1 | 67.236 |
| Probable ATP-dependent RNA helicase DDX17 | DDX17 | 65.50635 |
| 14-3-3 protein zeta/delta | YWHAZ | 64.6265 |
| Serine/threonine-protein phosphatase PGAM5, mitochondrial | PGAM5 | 64.3255 |
| Nucleolin | NCL | 63.2696 |
| Nucleoprotein TPR | TPR | 62.2519 |
| 14-3-3 protein eta | YWHAH | 59.842 |
| Enhancer of rudimentary homolog | ERH | 58.8375 |
| Protein FAM98A | FAM98A | 58.75631 |
| Putative 60S ribosomal protein L39-like 5;60S ribosomal protein L39 | RPL39P5;RPL39 | 56.28695 |
| Transcriptional activator protein Pur-beta | PURB | 55.112 |
| La-related protein 1 | LARP1 | 54.92573 |
| 60S ribosomal protein L8 | RPL8 | 54.7345 |
| Caprin-1 | CAPRIN1 | 54.059 |
| Ataxin-2 | ATXN2 | 53.36545 |
| RNA-binding protein FUS | FUS | 50.848 |
| Transcription factor 20 | TCF20 | 49.307 |
| Interleukin enhancer-binding factor 2 | ILF2 | 47.636 |
| Serine/arginine-rich splicing factor 1 | SRSF1 | 46.751 |
| Ras GTPase-activating protein-binding protein 2 | G3BP2 | 46.20865 |
| Double-stranded RNA-specific adenosine deaminase | ADAR | 44.8955 |
| Cleavage and polyadenylation specificity factor subunit 1 | CPSF1 | 43.576 |
| Zinc finger protein 638 | ZNF638 | 42.5365 |
| YTH domain-containing family protein 1 | YTHDF1 | 42.213 |
| Dual specificity mitogen-activated protein kinase kinase 1 | MAP2K1 | 42.15325 |
| Ras-related protein Rab-6B;Ras-related protein Rab-6A;Ras-related protein Rab-39A | RAB6B;RAB6A;RAB39A | 41.85205 |
| ELAV-like protein 1 | ELAVL1 | 41.432065 |
| Cellular nucleic acid-binding protein | CNBP | 41.149 |
| Intracellular hyaluronan-binding protein 4 | HABP4 | 40.6038 |
| Leukocyte receptor cluster member 1 | LENG1 | 40.4715 |
| Ribosome-binding protein 1 | RRBP1 | 40.4069 |
| Small nuclear ribonucleoprotein Sm D3 | SNRPD3 | 39.5835 |
| Serine/arginine-rich splicing factor 9 | SRSF9 | 38.7499 |
| Splicing factor 1 | SF1 | 38.615 |
| Myosin light polypeptide 6 | MYL6 | 38.40425 |
| Serine/arginine-rich splicing factor 7 | SRSF7 | 37.389505 |
| Calcium-binding mitochondrial carrier protein Aralar2 | SLC25A13 | 36.46795 |
| S-adenosylmethionine synthase;S-adenosylmethionine synthase isoform type-2 | MAT2A | 36.35585 |
| Protein FAM98B | FAM98B | 36.06 |
| 14-3-3 protein theta | YWHAQ | 34.8715 |
| ATP-dependent RNA helicase A | DHX9 | 34.66 |
| La-related protein 4B | LARP4B | 34.1545 |
| Splicing factor, proline- and glutamine-rich | SFPQ | 33.729 |
| Putative ATP-dependent RNA helicase DHX30 | DHX30 | 32.336 |
| YTH domain-containing family protein 2 | YTHDF2 | 31.4737 |
| OTU domain-containing protein 4 | OTUD4 | 31.4035 |
| Chromatin target of PRMT1 protein | CHTOP | 30.772 |
| THO complex subunit 4 | ALYREF | 30.2978 |
| Polyadenylate-binding protein 2 | PABPN1 | 29.7555 |
| Histone H1.4 | HIST1H1E | 28.801 |
| Transcriptional repressor NF-X1 | NFX1 | 28.733 |
| Non-POU domain-containing octamer-binding protein | NONO | 28.685275 |
| 60S ribosomal protein L7 | RPL7 | 28.502405 |
| 60S ribosomal protein L13a | RPL13A | 28.37565 |
| Serine/arginine repetitive matrix protein 1 | SRRM1 | 27.98 |
| Spectrin beta chain, non-erythrocytic 1 | SPTBN1 | 27.9115 |
| Heterogeneous nuclear ribonucleoprotein A0 | HNRNPA0 | 26.54565 |
| 60S ribosomal protein L27 | RPL27 | 26.4367 |
| Fragile X mental retardation protein 1 | FMR1 | 26.05015 |
| Tudor domain-containing protein 3 | TDRD3 | 25.899 |
