# Supplementary material for: KSR2-14–3-3ζ complex serves as a biomarker and potential therapeutic target in sorafenib-resistant hepatocellular carcinoma
Source: Biomark Res. 2022 Apr 25;10:25. doi: 10.1186/s40364-022-00361-9 (PMC9036720; doi:10.1186/s40364-022-00361-9)
Supplement: Supplementary file 2 — Additional file 2. [file 40364_2022_361_MOESM2_ESM.pptx]

## Slide 1
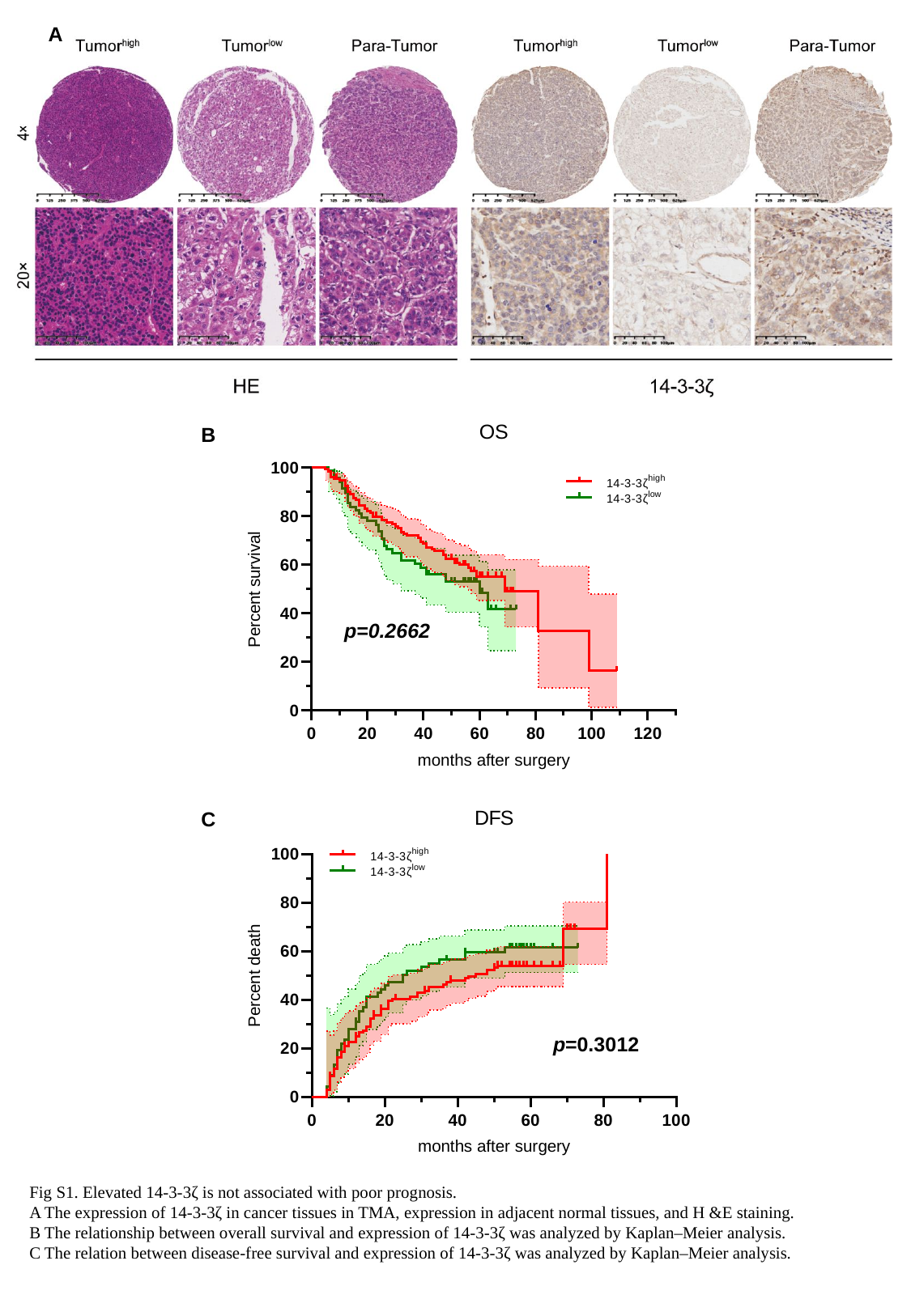

A
B
C
Fig S1. Elevated 14-3-3ζ is not associated with poor prognosis.
A The expression of 14-3-3ζ in cancer tissues in TMA, expression in adjacent normal tissues, and H &E staining.
B The relationship between overall survival and expression of 14-3-3ζ was analyzed by Kaplan–Meier analysis.
C The relation between disease-free survival and expression of 14-3-3ζ was analyzed by Kaplan–Meier analysis.
